# 2331 Community-informed adaptation of Group Well Child visits for limited English proficiency Latino families

**DOI:** 10.1017/cts.2018.244

**Published:** 2018-11-21

**Authors:** Rheanna Platt, Sarah Polk

**Affiliations:** Johns Hopkins University School of Medicine

## Abstract

OBJECTIVES/SPECIFIC AIMS: We propose to adapt a curriculum for group well-child care in order to (1) improve the experience of care for Latino immigrant families, (2) better address maternal psychosocial concerns impacting parenting and (3) teach parenting practices that promote healthy behaviors, and (4) improve LEP parent health literacy, engagement, and self-management in care. METHODS/STUDY POPULATION: This study is composed of a series of focus groups with 4 target populations: (1) The Johns Hopkins Bayview Children’s Medical Practice Latino Family Advisory Board (LFAB) (multiple meetings). The LFAB has been in existence since 2011, and has experience in iteratively adapting educational materials, both written and video, and in providing input on social work services and healthcare utilization. We will meet with the LFAB over the course of up to 8 meetings. During these meetings, the following themes will be discussed: (A) The concept of Group Well Child Care will be discussed and LFAB members will be asked about potential benefits and drawbacks of this format. (B) LFAB members will also be asked about group discussion topics that should be prioritized. Study staff will both bring up a list of topics (feeding, sleep, development, behavior, parent stress) and ask for input on additional items that should be discussed. (C) Core components of the mothers and babies course, a group perinatal depression intervention originally developed with Latina mothers, will be presented and discussed with board members, who will be asked to prioritize the components for salience and perceived helpfulness as well as inclusion in the Group Visit Curriculum. Potential benefits and drawbacks of including components of this program will also be asked of LFAB members. Members will not be asked about their depressive symptoms. (2) Pediatric providers (including social workers, MDs, NPs, and RNs) (1 focus group) who agree to participate will also be asked about perceived benefits and drawbacks of the group well-child care model, topics that should be prioritized in the educational components, and the benefits and drawbacks of including components of a perinatal depression prevention program in the group well child visit curriculum. (3) Obstetric group visit providers—Obstetric providers of group prenatal care to LEP Latinas at JHBMC will be asked about the benefits and drawbacks of group prenatal care with their patient population, as well as topics perceived to be of relevance to the patient population based on their experience with group prenatal care and discussions that emerged during the course of the facilitated groups. (4) Obstetric group visit patients (3 focus groups) LEP Latina patients who have participated in at least 3 group prenatal visits will be invited to participate in focus groups exploring the aforementioned topics. The experience of discussing psychosocial issues, including maternal depressive symptoms, in the group visit format will be emphasized. RESULTS/ANTICIPATED RESULTS: One focus group with obstetric providers and has thus far been conducted. Obstetric providers reported that patients were very open in discussing prior experiences with postpartum depression, and discussed feelings of loneliness with their peers in this setting. Anxiety was also frequently discussed. History of domestic violence was discussed more frequently than current domestic violence. DISCUSSION/SIGNIFICANCE OF IMPACT: Group visits may represent an opportunity to more effectively address psychosocial concerns in Latinas. Work needs to be done to understand which topics are most effectively and appropriately addressed in the group Versus individual format.

